# Confocal Fluorescence Anisotropy and FRAP Imaging of α-Synuclein Amyloid Aggregates in Living Cells

**DOI:** 10.1371/journal.pone.0023338

**Published:** 2011-08-08

**Authors:** M. Julia Roberti, Thomas M. Jovin, Elizabeth Jares-Erijman

**Affiliations:** 1 Laboratory of Cellular Dynamics, Max Planck Institute for Biophysical Chemistry, Göttingen, Germany; 2 Departamento de Química Orgánica, Facultad de Ciencias Exactas y Naturales, Universidad de Buenos Aires, CIHIDECAR, CONICET, Buenos Aires, Argentina; University Medical Center Groningen, University of Groningen, The Netherlands

## Abstract

We assessed the intracellular association states of the Parkinson's disease related protein α-synuclein (AS) in living cells by transfection with a functional recombinant mutant protein (AS-C4) bearing a tetracysteine tag binding the fluorogenic biarsenical ligands FlAsH and ReAsH, The aggregation states of AS-C4 were assessed by *in situ* microscopy of molecular translational mobility with FRAP (fluorescence recovery after photobleaching) and of local molecular density with confocal fluorescence anisotropy (CFA). FRAP recovery was quantitative and rapid in regions of free protein, whereas AS in larger aggregates was>80% immobile. A small 16% recovery characterized by an apparent diffusion constant of 0.03–0.04 µm^2^/s was attributed to the dynamics of smaller, associated forms of AS-C4 and the exchange of mobile species with the larger immobile aggregates. By CFA, the larger aggregates exhibited high brightness and very low anisotropy, consistent with homoFRET between closely packed AS, for which a Förster distance (*R*_o_) of 5.3 nm was calculated. Other bright regions had high anisotropy values, close to that of monomeric AS, and indicative of membrane-associated protein with both low mobility and low degree of association. The anisotropy-fluorescence intensity correlations also revealed regions of free protein or of small aggregates, undetectable by conventional fluorescence imaging alone. The combined strategy (FRAP+CFA) provides a highly sensitive means for elucidating both the dynamics and structural features of protein aggregates and other intracellular complexes in living cells, and can be extended to other amyloid systems and to drug screening protocols.

## Introduction

Pathological amyloidosis is characterized by the progressive formation in cells and organs of proteinaceous aggregates consisting predominantly of highly ordered, cross-β-sheet fibrils. A number of neurodegenerative diseases, notably α-synuclein (AS) in Parkinson's disease and Aβ peptide in Alzheimer's disease, feature such a deposition of small proteins [1]. The self-association of these proteins is progressive, involving the formation of soluble oligomers, protofibrils and finally insoluble mature fibrils. However, the factors precipitating these processes and the molecular species responsible for the progression of the disease are as yet unclear [2]–[6].

The large incidence of these and related disorders and their biological and medical impact have spurred extensive efforts to understand the underlying molecular mechanisms of neurotoxicity. In this regard, fluorescence microscopy is one of the most powerful methods for studying protein assembly, dynamics, and interactions in living and fixed cells, including processes that involve amyloid proteins [7]–[8]. For instance, the mobility of intracellular aggregated species has been reported by means of FRAP microscopy (fluorescence recovery after photobleaching microscopy) [8]–[10]. In addition, fluorescence anisotropy spectroscopy has been applied to the characterization of protein aggregation [11]–[19], and CFA (confocal fluorescence anisotropy) microscopy [20]–[22] allows the assessment of molecular motion and proximity, both of which undergo dramatic alterations during amyloid aggregation. In this work, we exploited a particular expression system [23] based on tetracysteine tags and biarsenical ligands for assessing amyloid formation in living cells by FRAP and CFA. The combination of these techniques constitutes a very versatile tool for elucidating both the structural and dynamic features of intracellular protein aggregates and oligomeric species.

**Figure 1:**
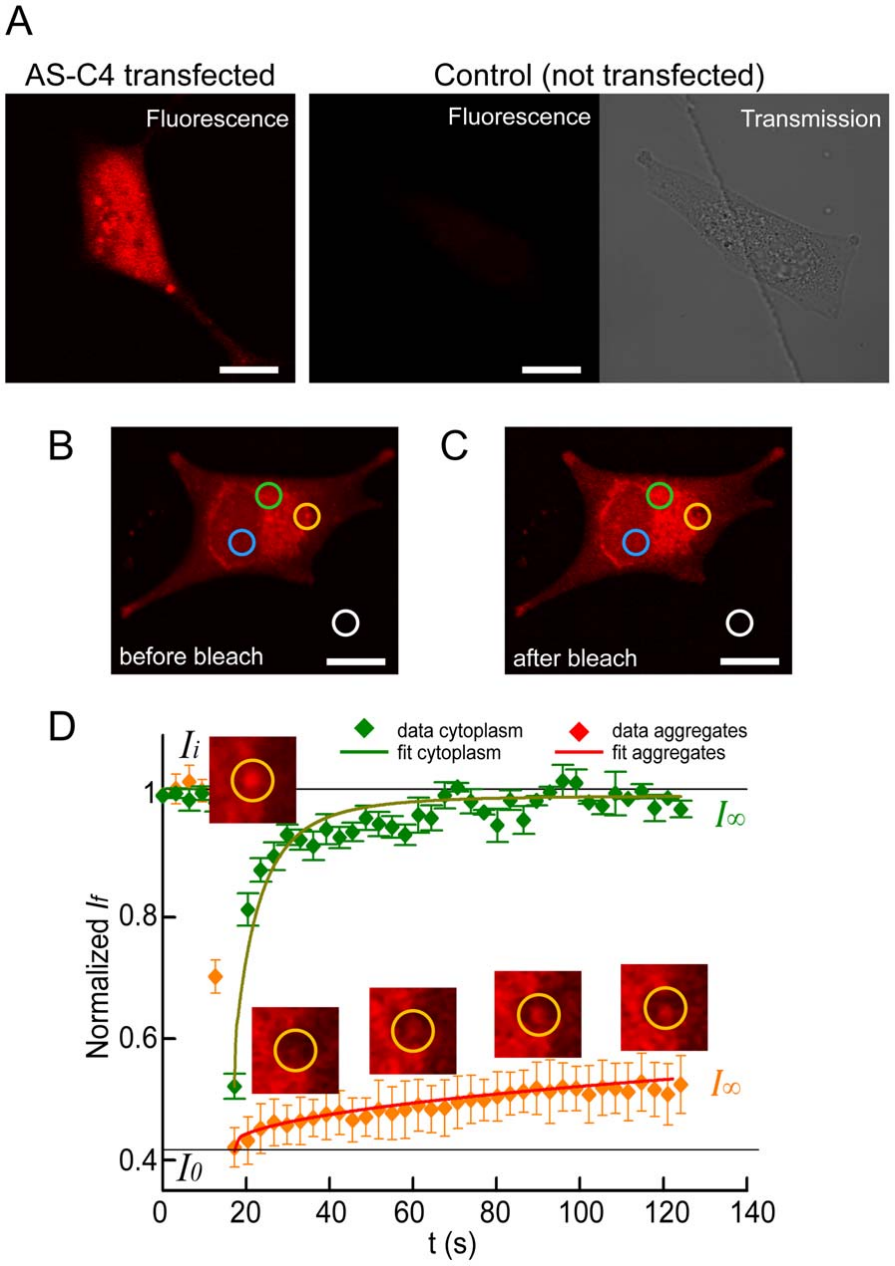
FRAP microscopy of fluorescently labeled AS-C4 aggregates in SH-SY5Y cells. (A) Cells were transiently transfected to express the biarsenical-binding AS-C4 version of AS, and ReAsH labeling allowed the identification of aggregated and non-aggregated regions with the protein, as opposed to non-transfected control samples that only exhibit a low background staining signal (the transmission image was included for a proper observation of the location of the cell). For a typical FRAP experiment performed on one cell, the regions of interest (*ROI*s, diameter ∼4 µm) before (B) and immediately after (C) photobleaching, are shown (colored circles): *ROI* with aggregates (yellow), *ROI* with no apparent aggregated AS-C4 (green), scanning-photobleaching control *ROI* (blue), and background *ROI* (white). (D) Normalized fluorescence recovery for the corresponding ROIs and their fits according to a simple monoexponential model. Fluorescence intensity values before (*I_i_*) and after (*I_0_*) photobleaching, and at the end of the experiment (*I_∞_*), are shown. The green arrow indicates the region of fast recovery for non-aggregated protein. The inset images correspond to the *ROI* with aggregated protein before photobleaching (*t* = 0 s), immediately after photobleaching (*t* = 17 s) and post-bleaching at *t* = 60, 90 and 120 s. Data shown as means ± standard errors (sample set *n* = 5).

We applied FRAP and CFA in combination to study the aggregation of the 140-aa AS protein in the SH-SY5Y [24] human neuroblastoma cell line commonly employed in amyloid studies. For this purpose, we employed a recombinant version of AS bearing a small tetracysteine tag (AS-C4) [25] capable of binding fluorogenic biarsenical compounds such as FlAsH and ReAsH. This expression system leads to highly stable and fluorescent protein complexes that retain the biophysical and biochemical properties of *wild-type* AS both *in vitro* and in living cells [25]. AS-C4 readily forms amyloid aggregates, as confirmed by standard ThioflavinT detection and specific antibody staining [25]. Another key advantage of this tag is that it adds only 1.3 KDa to the 14.5 KDa of AS; i.e. the size of the protein is almost unaltered. To perform the live cell experiments, SH-SY5Y cells were transiently transfected so as to express AS-C4 and subsequently labeled with either FlAsH or ReAsH. Fluorescent AS-C4 aggregates were observed 72 h after biarsenical labeling (Figure 1). For the purpose of illustrating the various labeling possibilities, we show FRAP and CFA performed with ReAsH and FlAsH complexes, respectively. The CFA results were also correlated with bulk measurements performed on AS-C4-FlAsH aggregation *in vitro*.

## Materials and Methods

### Reagents

All reagents were of analytical grade (Sigma). FlAsH(EDT)_2_ and ReAsH(EDT)_2_ were synthesized in our lab.

### Preparation of cell samples

SH-SY5Y cells [24] were kindly provided by Björn Falkenburger (European Neuroscience Institute, Göttingen, Germany). Cells were cultured at 37°C in 5% CO_2_ in complete medium and were transiently transfected using 2 µg of plasmid DNA and 5 µl of Lipofectamine 2000 in Opti-MEM (Invitrogen) to express AS-C4. Control samples prepared without plasmid were used to estimate background signal from unspecific ReAsH labeling. Three days after transfection, cells were stained with either FlAsH or ReAsH, as previously described [25]. Briefly, cells were incubated with 1 µM FlAsH (or ReAsH)/10 mM EDT in Opti-MEM, for 2 h at 37°C and 5% CO_2_ in the dark. Unbound dye was subsequently removed by washing 6 times for 30 min with 100 mM EDT in complete medium.

### Confocal microscopy

The experiments were carried out with a Zeiss SLM510 Meta confocal microscope equipped with an UPlanApo 63×1.2 NA water immersion objective. FlAsH was excited with an Ar-ion laser at 488 nm, and detected with a 515 longpass filter. ReAsH was excited with a solid state 532 nm laser and detected with a 585 longpass filter.

For anisotropy measurements, polarizers were inserted in the excitation and emission light paths of a CLSM to simultaneously acquire the polarized images *I_vv_*, *I_vh_* [20]; the subscripts specify the orientation of the excitation and emission polarizers (horizontal, vertical) [20].

For FRAP measurements, the laser power scanning excitation was set to 3%. The bleaching of the region of interest (ROI, 20 pixels ∼4 µm in diameter) was performed at a 100% laser power. A temporal image acquisition sequence was created, consisting of 1) a pre-bleaching imaging step, followed by 2) a ROI photobleaching step, and then by 3) a post-bleaching image acquisition step. Pre-bleaching was carried out by acquiring 5 images of the cell of interest (total time, 3 s), after which an ROI containing an aggregate or non-aggregated AS-C4-ReAsH was photobleached with 20 laser iterations. Finally, 40 post-bleaching images were acquired (3 s). The images were background corrected. A low level of photobleaching due to image scanning at 3% laser power was corrected by selecting a small region outside the ROI and estimating the average intensity of each frame relative to the initial frame.

Image processing in the case of the anisotropy and FRAP measurements was performed with DIPimage toolbox for Matlab (TU Delft, Holland) and with ImageJ (http://rsb.info.nih.gov).

### FRAP analysis

Fluorescence recovery curves were constructed from the total intensity fluorescence values in the region of interest for each frame of the temporal sequence, corrected for background and laser scanning bleaching, and normalized to the initial value (*F_i_*).

The mobile fraction associated to each ROI (*F_M_*) was estimated from the fluorescence intensity values before photobleaching (*F_i_*), immediately after photobleaching (*F_0_*), and at the end of the experiment (*F_∞_*), according to Equation E1:
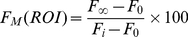
(1)


The recoveries of fluorescence of the aggregated and non-aggregated proteins were fitted to a monoexponential model.

### Anisotropy maps and image analysis

Confocal anisotropy maps were constructed from the polarized images using DIPimage, according to the following expression for *r* (Equation E2):
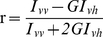
(2)


where G is a correction factor accounting for instrumental differences in the detection efficiencies of the polarizations. G was estimated by measuring the response of a depolarized 0.1 µM fluorescein solution deposited in a coverslip-slide assembly, according to Equation E3:
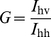
(3)


The pixel-by-pixel fluorescence and anisotropy values were mapped as 2-D histograms of fluorescence intensity *I_f_* vs. *r*. The correlation between *I_f_* and *r* was performed by establishing threshold values for each parameter, thus defining four quadrants in the fluorescence image distinguishing local areas with aggregates from those lacking aggregated protein, according to the information provided by *I_f_* (*high* values suggesting aggregation), and *r* (*low* values suggesting aggregation). The threshold value for *I_f_* was estimated as follows. We first measured the AS-C4-FlAsH fluorescence intensity in a region of the cell without aggregates and constructed a histogram, which was fit with a Gaussian distribution to extract the mean intensity (I*) and standard deviation (σ). From these values, we created a mask for AS-C4-FlAsH aggregates that included the pixels with intensities higher than the calculated mean intensity+5 times σ(*I_f_*>I*+5×δ). The data in Figure 1 are the means ± standard errors for a representative experiment (*n*>10 sample set, α = 0.05). The threshold value for *r* was set at *r* ≤ half the anisotropy of a solution of monomeric AS-C4-FlAsH (r*; see below for a description of the *in vitro* measurement procedure).

### Stationary fluorescence lifetime and anisotropy in AS aggregation assays *in vitro*

Stationary fluorescence intensity and fluorescence anisotropy determinations were performed in a Cary Eclipse spectrofluorometer (Varian, Australia). Fluorescence lifetime measurements were carried out with an IBH 5000 U fluorescence lifetime spectrometer (Glasgow, UK) equipped with a TBX-04-A picosecond photon detection module.

The aggregation of AS-C4 *in vitro* was determined on duplicate samples containing 500 µl of 100 µM AS-C4 (aggregation progress in time) or AS-C4-FlAsH (fluorescence lifetime and anisotropy evolution during aggregation) in 20 mM Na-Hepes buffer, pH 7.2, 100 mM NaCl, 0.02% NaN_3_ (and DTT 1 mM in the case of AS-C4). The samples were incubated at 37°C in glass vials with continuous magnetic stirring. Aliquots were withdrawn at different time-points. To confirm the existence of extent of energy homotransfer between like fluorophores in the labeled sample, an additional control solution consisting of a 1:10 FlAsH-labeled:unlabeled protein mixture was measured under the same conditions.

The time course of aggregation was monitored with a standard ThioflavinT assay on the AS-C4 samples [16], and plotted as the degree of fractional conversion (based on the initial and final signals) (α) as a function of time (*t*). The assays were performed in 50 mM Na-glycine buffer, pH 8.2, monitoring the increase emission at 480 nm (excitation wavelength 446 nm) due to amyloid formation. The data fit well to a sigmoidal model, with an aggregation half-lifetime of ∼76 h (the points in the curve are average values of the duplicates). AS-C4-FlAsH was not employed in these experiments because the presence of FlAsH interferes with the ThioflavinT determinations.

The temporal evolution of fluorescence lifetime (*τ*) and steady state anisotropy (*r*) associated with the progression of aggregation was measured on aliquots extracted from the AS-C4-FlAsH samples. The fluorescence lifetime was determined using a custom-made quartz microcuvette (optical path 0.25 mm) to allow measuring the undiluted labeled protein sample without internal filter effects excitation 485 nm, emission 530 nm) [A dilution of the aggregation mixture would be expected to disrupt the equilibrium between the transient species involved in the fibrillation process.] The emission decays were acquired under magic-angle conditions to eliminate polarization effects. Measurements were performed in time-correlated single-photon counting (TCSPC) mode using an IBH Data Station, v 2.1, with DAS6 software. The excitation source was a 485 nm pulsed NanoLED. The impulse response function (IRF) was obtained at the corresponding excitation wavelength using a Ludox scattering suspension. The data were analyzed with the DAS6 software.

Fluorescence polarization measurements were made using adjustable polarizers so as to acquire the parallel and perpendicular components of polarized emission (*I_vv_* and *I_vh_* respectively) with excitation at 485 nm and emission at 530 nm. The anisotropy was calculated according to Eqs. E2 and E3.

The fluorescence lifetimes of the labeled protein τ_AS-C4-FlAsH_, and the steady-state fluorescence anisotropies of the aggregation mixture normalized to that of monomeric AS-C4-FlAsH, were evaluated as a function of time.

## Results and Discussion

FRAP reports on the mobility of fluorescently labeled species by photobleaching a region of interest (*ROI*) and monitoring signal recovery over time *I(t)* [26]. From the recovery data, two parameters can be obtained [27]: the mobile fraction *F_M_*, and the associated diffusion constant D*. In order to assess these parameters for AS-C4, we carried out FRAP experiments on cells transfected to express AS-C4 and labeled with ReAsH (Figure 1A). In each sample imaged at the confocal microscope, we selected *ROI*s deemed to represent aggregated and non-aggregated ReAsH-labeled protein, respectively (Figure 1B,C).

First, the associated mobile fractions were determined from the final recovery intensities (Eq. E1). The recovery of the fluorescence in regions without aggregates was complete, whereas the fluorescence recovery in regions with aggregates was very limited, *F_M_* = 16±4% (Figure 1D). The almost complete recovery of fluorescence in the cytoplasm indicates that AS-C4 diffuses rapidly in that cellular compartment. However, the recovery was biphasic, consisting of a fast component (73%) occurring within a single imaging interval (∼3 s) and a subsequent slower component corresponding to the remaining 27%. A monoexponential fit to the slow phase yielded an apparent diffusion constant D* ∼0.08–0.15 µm^2^/s. The very fast (temporally unresolved) recovery corresponded presumably to free protein, for which the reported diffusion constant in solution is ∼10^2^ µm^2^/s [28]–[29]; a value for intracellular diffusion is not available but would be expected to be>10 µm^2^/s [30]. The slower recovery is attributable to membrane-bound protein and, possibly, microaggregates.

The situation differed substantially for fluorescence recovery in loci with obvious aggregates of AS-C4. In this case, the mobile fraction, estimated from the final fluorescence intensities, barely reached ∼16% (Figure 1D). That is, the majority of AS-C4 in the aggregates was virtually immobile, reflecting the inability of the protein in the core of such dense, compact structures to equilibrate with the cytoplasmic pool. We attributed the small yet finite recovery to the dynamics of smaller, associated and/or aggregated forms of AS-C4, and exchange of the mobile species with the larger aggregates. We fit the data to a monoexponential model, obtaining a diffusion constant D** ∼0.03–0.04 µm^2^/s. This value is significantly lower than D* and indicates that the AS aggregates remained essentially immobile. One should note that for FRAP determinations as those just described, AS-C4 is preferable to alternative expression probes of AS involving fusion with visible fluorescent proteins because the latter represent a two-fold increase in molecular mass [22], such that their own diffusion and FRET properties dominate. The insight into AS dynamics provided by FRAP finds an ideal counterpart in anisotropy imaging. Fluorescence anisotropy (*r*) provides information about parameters such as molecular rotation, size, shape and flexibility [20], [31]. In general, variations in *r* are directly correlated with changes in the size, and thus the rotational correlation time, of the species under study. However, *r* can also reflect the influence of Förster resonance energy transfer (FRET) between like species, a phenomenon denoted as homoFRET or energy migration FRET (*em*FRET) [20]–[21]. The occurrence of *em*FRET is manifested as a decrease of *r* by virtue of energy transfer from the original photoselectively excited molecules to randomly oriented, nearby molecules, resulting in an ensemble depolarization. Thus, anisotropy offers a powerful yet simple means for monitoring AS aggregation, inasmuch as it requires labeling with only a single marker and can be performed in living cells. Such a depolarization effect upon AS aggregation was previously established *in vitro* with experiments performed using an engineered AS-YFP chimera [22].

**Figure 2:**
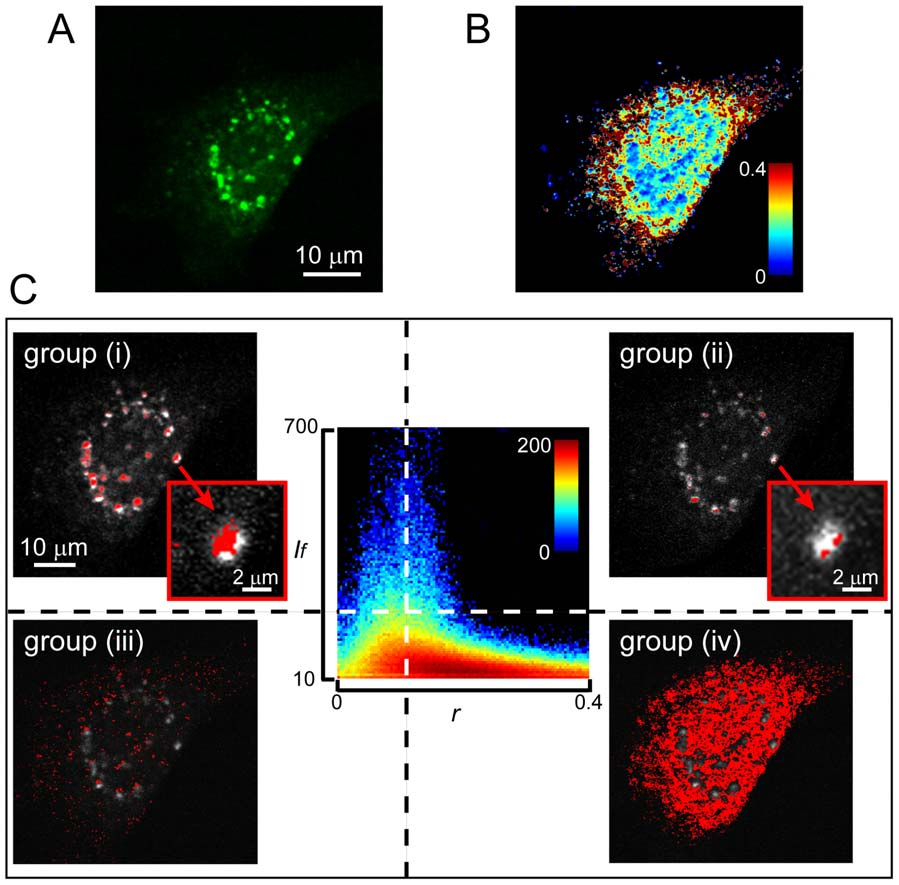
Confocal fluorescence anisotropy (CFA) microscopy of AS-C4-FlAsH aggregates in SH-SY5Y cells. (A) CFA image (*I_f_,*) and (B) associated fluorescence anisotropy (*r*) image. (C) 2D-histogram *I_f_* vs. *r*. The histogram values were sectioned using threshold values for *I_f_* and *r*, and the resulting (*I_f_*, *r*) pairs were backmapped on a pixel-by-pixel basis onto the fluorescence intensity image (red pixels). Four distinct groups were defined: (i) pixels with high *I_f_* and low *r* (top left), (ii) with high *I*_f_ and *r* (top right), (iii) low *I*_f_ and *r* (bottom left), and (iv) low *I*_f_ and high *r* (bottom right). The colored scale bar represents frequency (number of pixels). The insets in the images in groups (i) and (ii) show an example of an aggregate that displays high *I*_f_ and both low and high *r*, reflecting different dynamics of AS-C4 within its structure.

For the fluorescence anisotropy determinations, we employed two detection channels in the confocal microscope to simultaneously acquire images of the parallel and perpendicular components of polarized emission (*I_vv_* and *I_vh_*, respectively) in cells that expressed AS-C4 and were labeled with FlAsH. From these images, anisotropy maps associated with the total fluorescence intensity images *I_f_* were constructed [20] (Figure 2A,B).

The evaluation of the fluorescence intensity and anisotropy images revealed a distinct inverse correlation: most of the regions corresponding to aggregates, i.e. the very bright regions in the fluorescence image, showed very low *r* values (the dark-blue spots in the colormap). This relationship was further analyzed using 2D-histograms (Figure 2C), in which we set threshold values for *I_f_* (I_f_*) and *r* (r*) according to criteria relating these magnitudes to the presence of protein aggregation. In doing so, we segmented the histogram first into two regions demarcated by I_f_*, one for *I_f_* >I_f_* corresponding to AS-C4-FlAsH aggregates, and another for *I_f_* <I_f_*, homogeneously distributed values of no apparent aggregation. From the r* threshold (r* = ∼0.22 is the anisotropy value for monomeric AS-C4-FlAsH in solution), a second segmentation was made, one for low values of *r* (≤0.5·r*), and another for higher *r* values (>0.5·r*). In this manner, four quadrants were defined (Figure 2C). Each (*I_f_*, *r*) pair within each quadrant was backmapped onto its corresponding pixels in the original fluorescence intensity image (Figure 2C), yielding four distinct distribution maps.

**Figure 3:**
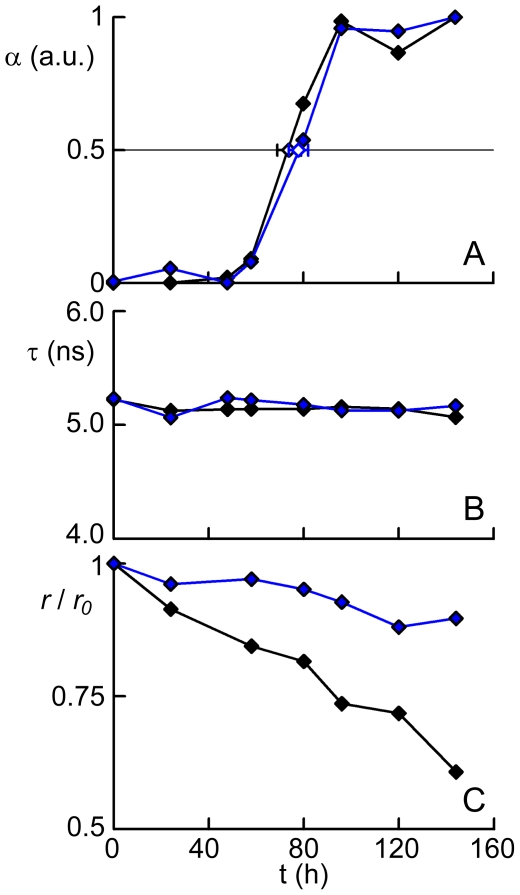
*In vitro* determination of anisotropy during AS-C4 aggregation. In each panel, black lines and diamonds correspond to AS-C4 in aggregation assays with ∼100% labeled protein, and blue lines and diamonds indicate AS-C4 in aggregation assays with a 1:10 FlAsH-labeled:unlabeled protein ratio. (A) AS-C4 normalized aggregation data measured by ThioflavinT emission. The black and blue empty diamonds indicate the mean half-lifetimes (*t*_1/2_) from a sigmoidal fitting of the data for each sample set, *t*_1/2_ = 74±4 h and 78±3 h, respectively. Error bars correspond to standard deviation of duplicate samples. (B) AS-C4-FlAsH fluorescence lifetime (τ) during the course of the aggregation assay. (C) Evolution of the steady-state fluorescence anisotropy during the aggregation assay.

The values from group (i) corresponded to AS-C4-FlAsH aggregates that were easily identified either in the fluorescence image (bright areas) or in the anisotropy map (dim areas with values of *r* decreased as much as 90% of the initial monomeric r* value). We ascribe this effect to *em*FRET between like FlAsH labels, a phenomenon supported by the following considerations. First, FRAP experiments showed that the aggregates were immobile, such that the depolarization could not have arisen from rapid movement of the labels in the densely packed structures. Second, the calculated Förster distance (*R_o_*) for FlAsH-FlAsH functioning as an *em*FRET donor-acceptor pair is∼5.3 nm. This value is larger than the previously reported (*R_o_* ∼3.9 nm) for the FlAsH-ReAsH donor-acceptor pair, a combination that already yielded positive *hetero*FRET signals in doubly-labeled AS-C4 intracellular aggregates due to the intimate association of protein monomers within the aggregated structures [25]. In another study based on the same AS-C4 probe and featuring luminescent nanoparticles (quantum dots) as initiators of aggregation, a coupled, sequential homoFRET-heteroFRET mechanism was invoked [32]–[33]. Finally, aggregation assays of AS-C4-FlAsH performed *in vitro* (Figure 3) showed a decrease in the steady-state anisotropy over time of ∼43% compared to the initial r* value for monomeric labeled protein (Figure 3C), as the bulk protein progressed from monomeric to oligomeric forms, resulting ultimately in amyloid fibrils (Figure 3A). The fluorescence lifetime (τ_AS-C4-FlAsH_) remained constant as *r* decreased (Figure 3B, C), in agreement with our previous findings [25] and those reported for the AS-YFP chimera [22]. The homoFRET-dependent depolarization is dimished upon separating the labels, as was achieved in aggregation assays in which the labeled:unlabeled protein ratio was adjusted to 1:10. Under this condition, the anisotropy decreased by only ∼10% whereas τ_AS-C4-FlAsH_ again remained constant (Figure 3C). The fact that the Thioflavin T signal reached a plateau value (Figure 3A) value while the anisotropy was still decreasing (Figure 3C) is indicative of a progressive condensation of the protein fibrils after their formation. This observation is yet another manifestation of the versatility of the AS-C4 construct.

CFA also provided a very sensitive tool for assessing molecular proximity±intimate association, a distinction that became apparent in the examination of *I_f_* and *r* in pixels belonging to group (ii). Here, CFA allowed the discrimination of some bright regions (based on the threshold established for *I_f_*) that appeared as aggregates, but whose associated *r* values were close to that of monomeric protein (*r_o_*), ruling them out as an aggregated species and suggesting an alternative attribution to planar, membrane-associated complexes [4]. Having a 2D rather than 3D character, the latter would be expected to exhibit lower FRET. An interesting observation was that some AS-C4-FlAsH aggregates backmapped in a singular fashion, displaying a large number of their pixels as belonging to group (i), and some pixels localized at the periphery of such structures as part of group (ii) and thus corresponding to mobile AS-C4-FlAsH (inset images in Figure 2C). A quantitative analysis of the pixel intensities from these aggregates showed that the ratio of the areas of pixels belonging to group (ii) relative to those of group (i) was 18±3% (*n* = 10, *P*<0.05). This value is in good agreement with the mobile fraction estimated from the FRAP experiments, suggesting that *r* is a reliable indicator distinguishing between mobile and immobile AS-C4-FlAsH.

Anisotropy imaging also revealed the presence of aggregates, not otherwise perceived due to virtue of their relatively low fluorescence, as shown by the pixels in group (iii) (Figure 2C). In this case, the low values of *r* indicated the presence of aggregates via *em*FRET. Finally, the values corresponding to group (iv) mapped to the large region of cytoplasm and nucleus with homogeneously distributed AS-C4-FlAsH, for which the values were equal or greater than those of the monomeric protein.

In the above experiments, the combination of FRAP and CFA imaging revealed both dynamic and structural features of AS-C4 aggregates in living cells. The FRAP experiments indicated that the aggregates were predominantly immobile compared to the homogeneously distributed protein in the cytoplasmic pool. In parallel, CFA positively identified aggregates as regions with *r* values significantly lower than that of the monomeric protein. Together, these findings established that the observed depolarization arose entirely from an *em*FRET process and not from other mechanisms dependent on protein mobility. We conclude that in the cellular context, AS forms multimeric and immobile structures typical of aggregated amyloid proteins, that might be at least partially associated with distinct, presumably membranous, compartments involved in native and/or aberrant functions [4]. Further studies will be aimed at determining the nature of such interactions, which may arise from AS self-association and incorporation into fibrillar (or prefibrillar) structures, and/or interaction with other proteins coaggregating with amyloid structures. e. g. tau and synphilin.

In summary, the combination of FRAP and CFA microscopies offers a robust and reliable means for the direct observation and characterization of the structure(s) and dynamics of amyloid aggregates and other protein complexes in living cells. This approach can be applied not only to the formation of such entities, but also to the inhibition or reversal of aggregation, an issue of immense biomedical interest in relation to drug screening and therapy of pathologic amyloidosis.
